# Quality of life, disability, and clinical variables in amyotrophic lateral sclerosis

**DOI:** 10.1590/0004-282X-ANP-2021-0201

**Published:** 2021-12-31

**Authors:** Mariana Asmar Alencar, Izaura Monique Moura da Silva, Stéfanie Marcelle Hilário, Marcela Ferreira de Andrade Rangel, Juliana Silva Abdo, Caroline Martins de Araújo, Leonardo Cruz de Souza

**Affiliations:** 1 Universidade Federal de Minas Gerais, Departamento de Fisioterapia, Belo Horizonte MG, Brazil. Universidade Federal de Minas Gerais Departamento de Fisioterapia Belo Horizonte MG Brazil; 2 Universidade Federal de Minas Gerais, Programa de Pós-Graduação em Neurociência, Belo Horizonte MG, Brazil. Universidade Federal de Minas Gerais Programa de Pós-Graduação em Neurociência Belo Horizonte MG Brazil; 3 Universidade Federal de Minas Gerais, Departamento de Medicina Interna, Belo Horizonte MG, Brazil. Universidade Federal de Minas Gerais Departamento de Medicina Interna Belo Horizonte MG Brazil

**Keywords:** Amyotrophic Lateral Sclerosis, Motor Neuron Disease, Quality of Life, Rehabilitation, Esclerose Amiotrófica Lateral, Doença dos Neurônios Motores, Qualidade de Vida, Reabilitação

## Abstract

**Background:**

Amyotrophic lateral sclerosis (ALS) is a motor neuron disease that results in a progressive increase in dysfunctions, limitations and restrictions over time, which can impact on quality of life (QoL). Therefore, expanding knowledge on QoL and possible factors associated with ALS can enable the development of actions to ensure greater wellbeing for the population.

**Objective:**

To investigate QoL in ALS and determine associations with demographic, functional and clinical aspects.

**Methods:**

Forty-five individuals with ALS (56.4±11.1 years) participated in the study. Demographic, clinical and functional aspects were investigated. Functioning and QoL were assessed using disease-specific tools (ALS Functional Ranting Scale-Revised/ALSFRS-R and ALS Assessment Questionnaire/ALSAQ-40). Fatigue was assessed using the Fatigue Severity Scale. Descriptive, correlation and stepwise multiple linear regression analyses were performed with the aid of the SPSS.

**Results:**

The mean ALSAQ-40 score was 279.0±118.3. QoL was significantly worse among women (p=0.001) and poor QoL was associated with the inability to walk (p=0.014), pain (p=0.021) and disease severity (p≤0.002). QoL was strongly correlated with the ALSFRS-R score (r=-0.82). Moderate to weak correlations were found for mobility [turning in bed (r=-0.62), locomotion (r=-0.33) and sit to stand (r=-0.40)], strength (r=-0.49), fatigue (r=0.35) and pain (r=-0.32) (p<0.03). The regression analysis revealed that the ALSFRS-R score (β=-0.76; p=0.00) and fatigue (β=0.20; p=0.04) were predictors of QoL.

**Conclusions:**

QoL was worse in women, older people, severe stages of ALS, patients with impaired mobility, those with a poorer physical performance and those who reported pain. Functional status and fatigue are predictors of QoL in ALS.

## RESUMO

**Antecedentes:**

Esclerose lateral amiotrófica (ELA) é uma doença do neurônio motor que resulta em um aumento progressivo das disfunções, limitações e restrições ao longo do tempo, o que pode afetar a qualidade de vida (QV). Ampliar o conhecimento sobre QV e os possíveis fatores associados permitirá o desenvolvimento de ações que garantam maior bem-estar.

**Objetivo:**

Avaliar a QV na ELA e verificar a associação com aspectos demográficos, funcionais e clínicos.

**Métodos:**

Participaram 45 indivíduos com ELA (5,4 ± 11,1 anos). Foram avaliados aspectos demográficos, clínicos, funcionais (ALS Functional Ranting Scale-Revised / ALSFRS-R), QV (ALS Assessment Questionnaire/ ALSAQ-40, respectivamente) e fadiga (Escala de gravidade da fadiga). Uma análise descritiva, correlação e regressão linear múltipla foram realizadas no programa estatístico SPSS.

**Resultados:**

Escore do ALSAQ-40 foi de 279,0 ± 118,3 para a população de ELA avaliada. Verificou-se pior QV significativa em mulheres (p=0,001), incapacidade de andar (p=0,014), dor (p=1,021) e com gravidade da doença (p≤0,002). QV correlacionou-se fortemente com ALSFRS-R (r =-0,82) e de moderada a fraca com mobilidade [virar na cama (r =-0,62), locomoção (r =-0,33) e sentar-se em pé (r=-0,40)], força (r=-0,49), fadiga (r=0,35) e dor (r=-0,32) (p<0,03). A análise de regressão encontrou ALSFRS-R (β=-0,76; p=0,00) e Fadiga (β=0,20; p=0,04) como preditores de QV.

**Conclusões:**

QV estava pior nas mulheres, em idosos, estágios de maior gravidade da ELA, na disfunção de mobilidade, no baixo desempenho e na dor. A funcionalidade e a fadiga na ELA são preditores de QV.

## INTRODUCTION

Amyotrophic lateral sclerosis (ALS) is a neurodegenerative disease characterized by progressive deterioration of the motor nervous system, involving the cortex, brainstem, and spinal cord^1^^,^^2^. The loss of lower motor neurons leads to muscle weakness, wasting, cramps and fasciculations, whereas the loss of upper motor neurons results in spasticity, clumsiness and brisk reflexes^1^^,^^2^. Extra-motor systems may also be involved in ALS, such as cognitive and behavioral disorders^1^^,^^2^.

The disease has a rapidly progressive course and causes cumulative disability in several domains, such as physical mobility, activities of daily living, independence, eating, communication, breathing and emotional reactions, eventually leading to death due to respiratory failure^3^^-^^5^. 

As there is no cure for ALS, the main aim of treatment is the prolongation of life, control of symptoms and implementation of supportive interventions to ensure quality of life (QoL) for as long as possible^6^. A multidisciplinary approach is essential and includes rehabilitation interventions with the goal of helping affected individuals reach their fullest potential and QoL despite the disabling disease^6^.

The clinical symptoms and the patterns of progression of ALS can have a negative impact on the lives of patients and their families. Thus, interest in studying QoL in ALS has increased in recent years^4^^,^^7^. However, there is no clear understanding of the perceptions of QoL in patients with ALS or what factors are related to QoL. Some studies have shown that the decline in physical function in this population does not seem to affect overall QoL and QoL does not change over time^7^^,^^8^. However, other studies show a significant deterioration of QoL throughout the course of the disease and report significant associations between the change in QoL and both physical and functional disabilities^4^^,^^9^. Moreover, some studies suggest that alleviating symptoms and improving QoL can affect the course of disease and survival^4^^,^^10^. 

Increasing knowledge on QoL and possible associated factors in ALS can allow the development of actions to ensure greater wellbeing and improve clinical care for individuals with ALS. As there is no consensus in the literature on this issue, the aim of the present study was to assess QoL in ALS and to investigate whether demographic (e.g., age, sex), clinical (e.g., initial presentation, severity of the disease, pain) and functional (e.g., mobility, physical performance) factors are associated with QoL. 

## METHODS

### Setting and participants

An observational cross-sectional study was conducted involving a convenience sample of 45 patients with probable or definite sporadic ALS, according to the Awaji criteria^11^. The patients were recruited from the ALS outpatient clinic of the university hospital affiliated with Universidade Federal de Minas Gerais (UFMG). Eligible patients for the study had no history of neurological diseases such as stroke, spinal cord injury, Parkinson’s disease or signs and symptoms of frontotemporal dementia. All participants provided written informed consent. This study received approval from the UFMG institutional review board.

### Demographic and clinical characteristics

The following demographic and clinical data were collected: age, sex, medications, hospitalization, ventilation, pain (occurrence and intensity based on a numerical pain rating scale from 0 to 10) and mobility (sit to stand ability, turning in bed and walking). The following aspects specific to ALS were also collected: disease duration (years), site of onset of symptoms (limb or bulbar) and use of riluzole.

### Quality of life

QoL was assessed using Amyotrophic Lateral Sclerosis Assessment Questionnaire-40 (ALSAQ-40), which is specifically designed to evaluate the QoL of patients with ALS. The AALSAQ-40 addresses five domains that are normally compromised in motor neuron diseases: eating, communication, activities of daily living and independence, mobility, and emotional aspects. The score for each domain ranges from 0 to 100. The total score is determined from the sum of the domains and ranges from 0-500, with higher scores denoting worse QoL^12^.

### Functional assessment -ALS specific questionnaire

The Amyotrophic Lateral Sclerosis Functional Rating Scale-Revised (ALSFRS-R) was used for functional assessment^13^. The ALSFRS-R addresses 12 items (speech, salivation, swallowing, handwriting, cutting food and handling utensils, dressing and hygiene, turning in bed and adjusting bed clothes, walking, climbing stairs, dyspnea, orthopnea and breathing insufficiency) that assess gross motor function, fine motor function, and bulbar and respiratory involvement. Each function is scored from 0 to 4 points and the total ranges from zero (maximum disability) to 48 (no disability). In this study, we also categorized the ALSFRS-R score into three stages of severity: mild (score: 37-48), moderate (score: 25-36) and severe (score: 0-24) ^14^^,^^15^.

### Muscle strength

Muscle strength was measured bilaterally using the Medical Research Council (MRC) scale, which is scored on a scale from 0 (absence of movement) to 5 (normal strength) points. Four upper limb muscles (wrist extension, wrist flexion, elbow flexion and shoulder abduction) and four lower limb muscles (ankle dorsiflexion, plantar flexion, hip extension and flexion) were considered. The score of the 16 muscle groups produced an overall strength score ranging from 0 to 80 points^4^^,^^16^^,^^17^.

### Physical performance

Physical performance was assessed using the Short Physical Performance Battery (SPPB), which involves three objective tests of lower body function: 1) timed three-meter walk at a normal pace using the best of two times; 2) time required to stand up from a chair five times as fast as possible; and 3) three individual tests of standing balance, which include a side-by-side stance, semi-tandem stance, and tandem stance, with the maximum score given for successfully standing for 10 seconds during each individual test. Each task is scored from zero to 4 points. The scores on the three tests are summed to give the total, which ranges from 0 to 12, with higher scores indicating a higher level of functioning^18^.

### Fatigue

The Fatigue Severity Scale (FSS) was used to assess fatigue. The FSS is a self-report questionnaire with nine items addressing the severity of fatigue in daily life. Each statement is scored from 1 (strong disagreement) to 7 (strong agreement). The FSS score is obtained by calculating the average of all items and a score ≥ 4 indicates the presence of fatigue^19^.

### Statistical analysis

Descriptive analysis was performed using frequency and measures of central tendency and dispersion according to the characteristics of each variable of interest. The normality of the data was tested using the Shapiro-Wilk test. 

We performed three set of analyses. First, we compared QoL between the following subgroups of patients: male vs female, site of onset (spinal vs bulbar), severity of the disease (mild, moderate and severe, according to ALSFRS-R), pain, ambulation, turning in bed and sit-to-stand capacity. Student’s t-test for independent samples or ANOVA with turkey post-hoc test was used to compare the difference in QoL between subgroups. 

Secondly, we investigated correlations between the score on ALSAQ-40 and demographic and clinical variables. Pearson or Spearman correlation tests were used to explore these correlations. 

Lastly, a multiple linear regression was used to determine independent variables predictive of the response variable (QoL). The final model was created using the stepwise forward method. Variables correlated to QoL, with clinical relevance, were used as the independent variables (age, ALSFRS-R, fatigue, pain and muscle strength). Multicollinearity was tested when selecting the independent variables. All analyses were performed with the SPSS 20.0 program and the level of significance was set at 5% (p < 0.05).

## RESULTS

### Demographic and clinical characteristics of ALS participants

Forty-five patients with ALS (56.44 ± 11.07 years) participated in the present study. Median of disease duration was three years (min-max: 0-6). Most participants were male (60%) and had initial spinal presentation (82.2%). Regarding QoL, the mean total ALSAQ-40 score was 279.03 ± 118.26. The demographic and clinical characteristics of the participants as well as the scores of the QoL domains (mobility, activities of daily living, eating, communication and emotional aspects) are shown in Table 1.

**Table 1.** Demographic and clinical characteristics of participants.

Characteristics		ALS (n = 45)		
		n (%)	mean ± SD	median; min-max
Age (years)			56.4 ± 11.1	
Sex (male)		27 (60)		
Disease duration (years)				3; 0-6
Site of symptom onset	Limb	37 (82.2)		
	Bulbar	8 (17.8)		
Pain		24 (53.3)		
Pain intensity			5.6±1.9	
N of medications				3; 0-12
Riluzole use		32 (71.1)		
Non-invasive ventilation		9 (20)		
Tracheotomy/mechanical ventilation		0 (0)		
Mobility	*Sit-to-stand* Able to perform independently Unable to perform without assistance	20 (44.4) 25 (55.6)		
	*Turning in bed* Able without assistance Unable without assistance	24 (53.3) 21 (46.7)		
	*Ambulation capacity* Able to walk Unable to walk	30 (66.7) 15 (33.3)		
ALSFRS-R total score			28.2 ± 10.6	
ALS severity level	Mild	13 (28.9)		
	Moderate	15 (33.3)		
	Severe	17 (37.8)		
Muscle strength (global score)			37.2 ± 15.9	
SPPB (physical performance)				1; 0-13
Fatigue			3.42 ± 2.1	
ALSAQ-40 domains	Mobility		72.72 ± 30.79	
	ADL		68.06 ± 34.34	
	Eating		36.12 ± 37.68	
	Communication		50.47 ± 44.65	
	Emotion		51.67 ± 31.60	
Total			279.03 ± 118.26	

ALS: amyotrophic lateral sclerosis; ALSFRS-R: Amyotrophic Lateral Sclerosis Functional Rating Scale-Revised questionnaire; SPPB: Short Physical Performance Battery; ALSAQ-40: Amyotrophic Lateral Sclerosis Assessment Questionnaire-40; Mobility: physical aspects and mobility; ADL: activities of daily living and independence; Eating: eating and drinking; communication: communication skills; emotion: emotional aspects.

### Subgroup comparisons

The data regarding the QoL comparisons in different subgroups (sex, site of onset, ALS severity level, pain, walking, turning in bed and sit-to-stand capacity) are shown in Table 2. QoL was significantly worse among women (p = 0.001), individuals who were unable to walk (p = 0.014), unable to turn in bed without assistance (p = 0.000), unable to change from a siting to a standing position (p = 0.000) and in those who reported pain (p = 0.021). QoL differed among the different stages of the disease (p ≤ 0.002) in all comparisons between groups, with worse QoL in more severe stages of the disease. No statistically significant difference was found in QoL regarding the site of disease onset (bulbar/limb). 

**Table 2.** QoL according to different clinical and functional characteristics.

Variable	n	ALSAQ-40 score	p-value	
Sex	Male	27	231.5 ± 97.6	0.001*
	Female	18	350.25 ± 112.5	
Site of symptom onset	Limb	37	266.5 ± 125.2	0.140
	Bulbar	8	335.9 ± 70.4	
ALS severity level	Mild	13	155.3 ± 84.94	<0.002*
	Moderate	15	275.4 ± 43.7	
	Severe	17	376.9 ± 93.5	
Pain	Yes	24	316.54 ± 120.5	0.021*
	No	21	236.15 ± 102.2	
Ambulation capacity	Yes	30	248.9 ± 109.7	0.014*
	No	15	339.14 ± 114.84	
Turning in bed capacity	Yes	24	208.11 ± 94.63	0.000*
	No	21	359.11 ± 94.6	
Sit-to-stand capacity	Yes	20	208.83 ± 102.78	0.000*
	No	25	335.17 ± 99.42	

ALSAQ-40: Amyotrophic Lateral Sclerosis Assessment Questionnaire-40; ALS: amyotrophic lateral sclerosis.

### Correlation analysis

QoL was strongly correlated with the ALSFRS-R score (r = -0.82; p = 0.000), indicating that individuals with more severe functional impairment had worse QoL. Older age was also correlated with poorer quality of life in individuals with ALS (r = 0.38; p = 0.010). Muscle strength (r = -0.49; p = 0.001), SPPB score (r = -0.40; p = 0.012), pain intensity (r = 0.32; p = 0.033) and fatigue (r = 0.35; p =0.021) were also correlated with the perception of QoL. Thus, it was found that lower strength, worse physical performance, higher pain intensity and fatigue were related to worse QoL in ALS. The correlations are shown in Table 3.

**Table 3.** Correlations between QoL and functional/clinical variables in sample of patients with ALS**.**

Variables	r	p-value
Age	0.38	p = 0.010*
Disease duration (years)	-0.12	p = 0.449
ALSFRS-R	-0.82	p = 0.000*
Muscle strength	-0.49	p = 0.001*
SPPB	-0.40	p = 0.012*
Pain intensity	0.32	p = 0.033*
Fatigue	0.35	p = 0.021*

QoL: Quality of life; ALS: Amyotrophic lateral sclerosis; r: correlation coefficient; ALSFRS-R: Amyotrophic Lateral Sclerosis Functional Rating Scale-Revised questionnaire; SPPB: Short Physical Performance Battery.

### Multiple regression analysis

Multiple regression analysis was carried out to investigate whether age, ALSFRS-R, fatigue, pain and muscle strength could significantly predict QoL. The final multiple linear regression model included the ALSFRS-R score (β = -0.760; t = -8.06; p = 0.000) and fatigue (β = 0.200; t = 2.12; p = 0.041). The results of the regression analysis indicated that the model explained 68.9% of the variance and was a significant predictor of QoL [F(1.37) = 4.494, p ≤ 0.041] (Table 4). Functional status exerted a stronger influence, as the ALSFR-S score explained 76% of the variation in the ALSAQ-40 score.

**Table 4.** Predictors of QoL in ALS.

Predictors	R^2^	Adjusted R^2^	F(1.37)	Β	p-value
ALSFRS-R and Fatigue Model	0.689	0.673	4.494		<0.041
ALSFRS-R				-0.760	0.000
Fatigue				0.200	0.041

QoL: Quality of life; ALS: Amyotrophic lateral sclerosis; R^2^: coefficient of determination; F: F statistic; ALSFRS-R: Amyotrophic Lateral Sclerosis Functional Rating Scale-Revised questionnaire.

## DISCUSSION

In the present study, QoL was worse in women, older people, in those with severe stages of ALS, impaired mobility, poorer physical performance, and those who reported pain. Functional status and fatigue in ALS were predictors of QoL and functional status exerted a stronger influence. These findings confirm the importance of functional status to QoL in ALS^4^^,^^9^^,^^20^. As maintaining QoL during the course of the disease is important, clinicians must be aware of the factors associated with poorer QoL to provide optimal care^20^. 

The neurobiological nature of ALS leads to functional loss over time. The outcome is progressive decline, which results in an increasing number and severity of impairments, functional limitations and disabilities that can exert an impact on QoL^21^. However, there is no consensus on the association between the progression of functional disability in patients with ALS and QoL. In the present study, an association was found between physical function and QoL. We found that the ALSFRS-R score explained 76% of the variation in the ALSAQ-40 score, which is in agreement with a previous association (75%) described by Prell et al*.*^9^. However, this result contrasts findings described in other studies^7^^,^^8^^,^^22^. A probable explanation for these divergent results is the difference in the type of tool (generic or disease-specific) used to assess QoL^4^^,^^7^^,^^22^. Studies employing a generic instrument to assess QoL in ALS have indicated the maintenance of QoL despite the decline in physical function^7^^,^^8^^,^^22^, whereas studies using a disease-specific instrument report a reduction in QoL with the increase in physical impairment in ALS^4^^,^^9^. Disease-specific QoL instruments seem to better identify characteristics related to ALS and may be more sensitive to disease-related changes. The use of disease specific instruments may increase the magnitude of prediction compared to generic instruments^9^. 

We also compared QoL regarding different aspects of functional capability, which is a novel information in the literature. Activities such as turning in bed, ambulation capacity and going from the sitting to standing position are extremely important aspects of mobility that ensure independence^20^. The inability to perform these tasks without assistance is related to worse QoL in ALS. Besides the impact on independence, a reduction in mobility can cause sleep disorders as well as pain due to immobilization^23^. Thus, maintaining mobility for as long as possible may be beneficial to patients with ALS. To complement the assessment of function measured by a self-report questionnaire, we investigated aspects of mobility using the SPPB, evaluating the execution of different tasks or actions (walking three meters, sit-to-stand five times and balance)^18^. Thirty of the 45 participants in the sample were able to perform this battery of tests and a correlation was found between the total SPPB score and QoL. This finding further strengthens the previous result that functional capacity contributes to QoL^20^. This correlation was found both by a self-report measure and by a measure of activity performance. However, further research is required to better understand the influence of maintaining mobility on QoL. 

Fatigue was an independent variable contributing to the final multiple linear regression model. The correlation between fatigue and quality of life has been found in previous studies^24^^-^^26^, but no studies were found in which fatigue was considered a predictor of QoL. Fatigue is a common and debilitating symptom in individuals with ALS and can cause distress as well as a reduction in physical function^24^^-^^26^. Fatigue in ALS probably has a multifactorial etiology and appears to be experienced primarily as a general feeling of whole-body tiredness as well as physical aspects (reversible motor weakness)^24^^,^^26^. Despite the adverse effects of fatigue, symptoms can be minimized through effective management^24^. Therefore, recognizing the signs of fatigue, identifying factors that worsen symptoms, and learning ways to maintain energy can have an impact on the QoL of patients with ALS. Despite the complexity and importance of fatigue in ALS, few studies are found in the literature. 

For a more in-depth investigation of QoL in ALS, this study also compared QoL between the sexes and sites of onset (spinal vs. bulbar) as well as among severity categories (mild, moderate, and severe, according to ALSFRS-R). We also investigated correlations between the ALSAQ-40 score and age, disease duration, and muscle strength. 

We found that women had significantly worse QoL scores compared to men. In contrast, other studies found that sex had no influence on QoL in patients with ALS^4^^,^^22^. The interaction between gender and clinical features in ALS is complex and related to disease onset, age at onset, the clinical course of the disease, and different biological responses, which may affect disease impairment and QoL^27^. However, the influence of sex should be further investigated. The negative correlation found between aging and QoL is in agreement with data reported in a previous study^22^. Functional decline is common in older people and physical impairment is exacerbated when aging is combined with a neurodegenerative disease, perhaps determining an early worsening of both disability and QoL^28^. Some studies found age to be a prognostic factor for survival and physical disability in ALS^28^^,^^29^. 

No difference in QoL was found in relation to the site of symptom onset (bulbar or limbs). Some studies have reported a worse prognosis with bulbar onset, with a higher rate of disability and, consequently, worse QoL^4^^,^^22^^,^^30^. In the present study, however, no difference was found between bulbar and limb onset. This divergence may be explained by factors such as different sample characteristics, differences in the age of the participants^4^^,^^30^, the sample size for bulbar onset in the present study, and the different instruments employed for the assessment of QoL^22^^,^^30^. 

A significant reduction in QoL was found with the increase in the severity of ALS. This result is in agreement with findings described in the literature^4^^,^^9^. QoL worsens with the progression of impairments. Despite the progressive physical impairment that occurs throughout the course of the disease and the correlation between the severity of ALS and poorer QoL, we found no significant correlation between QoL and disease duration. A similar finding is described in a previous study^22^. Disease duration and its progression varies greatly among individuals with ALS. The average life expectancy is two to five years after diagnosis. However, many people can live with the disease for longer than five years and with different degrees of impairment^1^. In addition, we often encounter the problem of inaccurate information regarding the onset of disease. Therefore, disease duration is not the best information to guide rehabilitation and the therapeutic plan.

Pain was correlated with a poor QoL. Associations between QoL and pain have been identified in previous studies^16^^,^^31^. Pain is a frequent symptom in ALS and is largely overlooked in clinical settings, despite the stressful experiences and negative impact it may cause^16^^,^^31^. Clinicians should be aware that pain exerts a negative impact on QoL and should be managed effectively throughout the course of the disease.

We found a moderate significant negative correlation between muscle strength and QoL in ALS, which is in agreement with data reported in the literature^4^. Muscle weakness and atrophy are considered the first signs of ALS and are usually the reason to see a doctor. Initial muscle weakness usually occurs in isolated muscles, followed by progressive, generalized atrophy and weakness, which have an adverse effect on functioning and wellbeing^20^. Therefore, monitoring changes in strength may provide information about functional decline and, consequently, a reduction in QoL.

We acknowledge certain limitations of the present study. The first is the cross-sectional design, which does not allow predicting QoL changes throughout the progression of the disease. Thus, longitudinal studies are needed to investigate the influence of disease progression on QoL. Another limitation was the use of a convenience sample of individuals recruited from a specialized ALS clinic. Although the outpatient clinic is a public reference center in a large state in Brazil, the use of a convenience sample limits the generalizability of results. Another important limitation was the fact that we did not evaluate depression, anxiety, or other mood disorders in the patients or caregivers, which are issues that may be related to QoL. 

The present study highlights the importance of functional status as a key variable that influences QoL in ALS. The diversity of factors shown to be related to QoL in ALS underscores the importance of the implementation of multidisciplinary care. As ALS is an incurable neurodegenerative disease and QoL should guide care management, healthcare providers should pay close attention to secondary symptoms (i.e*.*, pain and fatigue) and functional decline. 
